# Diabetes Mellitus among Patients with Acute Ischemic Stroke Admitted to the Department of Medicine in a Tertiary Care Centre

**DOI:** 10.31729/jnma.8394

**Published:** 2024-01-31

**Authors:** Parash Rayamajhi, Janak Khadka, Pravesh Bhattarai, Anjana Bohaju, Apsara Adhikari, Deependra Mandal

**Affiliations:** 1 Department of Neurology, Kathmandu Medical College and Teaching Hospital, Sinamangal, Kathmandu, Nepal; 2 Kathmandu Medical College and Teaching Hospital, Sinamangal, Kathmandu, Nepal; 3 Sylhet Women's Medical College, Mirboxtula, Sylhet, Bangladesh

**Keywords:** *diabetes mellitus*, *glycated hemoglobin*, *ischemic stroke*

## Abstract

**Introduction:**

Diabetes mellitus is a major public health concern and a continuously growing chronic disease worldwide. Diabetes mellitus is one of the modifiable, independent, and major risk factors of ischemic stroke. This study aimed to find the prevalence of diabetes mellitus among patients with acute ischemic stroke admitted to the Department of Medicine in a tertiary care centre.

**Methods:**

This descriptive cross-sectional study was conducted among patients with acute ischemic stroke admitted to the Department of Medicine from 19 July 2023 to 19 November 2023 after obtaining ethical approval from the Institutional Review Committee. Detailed clinical history, random blood sugar and glycated haemoglobin were used to define diabetes mellitus. A convenience sampling method was used. The point estimate was calculated at a 95% Confidence Interval.

**Results:**

Among 80 patients, diabetes mellitus was seen in 25 (31.25%) (21.09-41.41, 95% Confidence Interval). Among them, 19 (76%) had previously diagnosed and 6 (24%) had newly diagnosed diabetes mellitus. The poor glycemic control was seen in 11 (44%).

**Conclusions:**

The prevalence of diabetes mellitus among patients with acute ischemic stroke was found to be higher than in studies done in similar settings.

## INTRODUCTION

Diabetes mellitus (DM) is a group of metabolic diseases characterised by hyperglycemia resulting from defects in insulin secretion, insulin action or both.^1^ High systolic blood pressure (BP), high body mass index (BMI), high fasting blood glucose, ambient particulate matter pollution and smoking are the five leading risk factors of stroke.^2^ Diabetes mellitus causes vascular endothelial dysfunction, increased early-age arterial stiffness, and systemic inflammation along with an increased risk of encountering other risk factors for stroke.^3^

The incidence of stroke has been increasing in lower- middle-income countries (LMIC) such as Nepal. LMIC accounts for >75% of global stroke-related deaths and >80% of disability-adjusted life years (DALY).^4^ Very few studies have been done in Nepal regarding the prevalence of diabetes mellitus among ischemic stroke.

This study aimed to find the prevalence of diabetes mellitus among acute ischemic stroke patients admitted to the Department of Medicine in a tertiary care centre.

## METHODS

A descriptive cross-sectional study was conducted among acute ischemic stroke patients admitted to the Department of Medicine of Kathmandu Medical College and Teaching Hospital, Sinamangal, Kathmandu, Nepal from 19 July 2023 to 19 November 2023. The ethical approval was taken from the Institutional Review Committee of the same institute (Reference number: 16072023/05). Patients aged 18 years and above, who gave consent for the study and those who were diagnosed with acute ischemic stroke clinically and radiologically by magnetic resonance imaging (MRI) or non-contrast computed tomography (NCCT) scan were included in the study. Patients with hemorrhagic stroke, epilepsy, and subdural hematoma and those who did not give consent for the study were excluded from our study. A convenience sampling method was used. The sample size was calculated using the formula:


$$\begin{array}{ll} \text{n} & ={\text{Z}}^{2}\times \frac{\text{p}\times \text{q}}{{\text{e}}^{\text{2}}}\\ & =1.96^{2}\times \frac{0.25\times 0.75}{0.10^{2}}\\ & =72 \end{array}$$

Where,

n = minimum required sample size
Z = 1.96 at 95% Confidence Interval (CI)
p = prevalence of diabetes taken as 25% from the previous study^5^
q = 1-p
e = margin of error, 10%

The calculated sample size was 72. However, 80 patients were included in our study.

Information was collected from patients and their family members. Demographic profiles such as age and sex were recorded from all the patients along with the history like existing diabetes mellitus, medication used for diabetes etc. Random blood sugar (RBS), and glycated hemoglobin (HbAIC) were investigated for every acute ischemic stroke patient. A newly detected diabetes mellitus was diagnosed for patients who have RBS ≥200 mg/dL with clinical symptoms and HbAIC ≥6.5% as outlined in diagnostic guidelines of the American Diabetic Association.^1^

Data were entered and analyzed using IBM SPSS statistics version 21.0. The point estimate was calculated at 95% CI.

## RESULTS

Among 80 patients with acute ischemic stroke, diabetes mellitus was seen in 25 (31.25%) (21.09-41.41, 95% CI). Previously diagnosed diabetes mellitus was seen in 19 (76%) and newly diagnosed in 6 (24%) patients. The mean age of patients with acute ischemic stroke presenting with diabetes mellitus was 65.96±10.28 years. Age-wise distribution depicted that 17 (68%) patients with diabetes mellitus belonged to the age group of ≥60 years and 8 (32%) patients to the 40-59 age group. There was male predominance with male patients being 13 (52%) (Table 1).

**Table 1:** Demographic characteristics of acute ischemic stroke patients with diabetes mellitus (n= 25).

Age group (years)	n (%)
18-39	-
40-59	8 (32)
≥60	17 (68)
**Gender**	
Male	13 (52)
Female	12 (48)

Hypertension was seen in 19 (76%) as the commonest co-morbidity for acute ischemic stroke followed by dyslipidemia seen in 14 (56%) (Table 2).

**Table 2:** Co-morbidities among acute ischemic stroke patients with diabetes mellitus (n= 25).

Co-morbidities	n (%)
Hypertension	19 (76)
Dyslipidemia	14 (56)
Atrial fibrillation	4 (16)
Myocardial infarction	3 (12)
Smoking	12 (48)

Among the patients with diabetes mellitus, 11 (44%) have HbA1C ≥7% with poor glycemic control (Table 3).

**Table 3:** Glycemic control among acute ischemic stroke patients with diabetes mellitus (n= 25).

HbA1C (%)	n (%)
≥7	11 (44)
<7	14 (56)

## DISCUSSION

The prevalence of diabetes mellitus was 25 (31.25%) in our study. According to previous research, the prevalence of diabetes mellitus in Nepal is estimated to be 10%.^6^ A study conducted in Nepal showed the prevalence of 28.53% .^7^ Some studies also demonstrated that the presence of diabetes mellitus in ischemic stroke is associated with worse outcomes, poor neurological and functional outcomes, longer duration of hospital stay, and a higher rate of recurrence of stroke.^8^ In contrast, our results have a higher prevalence than reported in previous research from Nepal Medical College and Teaching Hospital.^9^ Higher prevalence of diabetes mellitus in stroke patients in our study may be due to differences in the study period and a combination of factors that affect diabetes such as rapid urbanization, changing lifestyles, unhealthy diets, and increasing life expectancy. The higher prevalence in our study suggests that neurologists in Nepal have to manage a large population of ischemic stroke with diabetes. It also shows higher complications and bad prognosis of stroke patients in Nepal as diabetes mellitus increases complications in stroke patients.

In the present study, the mean age in Diabetic stroke patients was 65.96±10.2 years which was supported by the findings of a recent meta-analysis.^10^ In contrast, the mean age of ischemic stroke patients with diabetes mellitus was higher in Western countries.^11^ This may be due to inadequate screening and controlling of modifiable risk factors in our country where the poverty line of the population and inaccessibility to health services are still on the higher side.

In this study, the prevalence of diabetes mellitus in ischemic stroke patients was higher in males than in females. This is similar to the result of the study in Dhaka.^12^ Worldwide 17.7 million more men have diabetes than women as men have greater insulin resistance, higher fasting glucose levels, and higher visceral fat mass than women.^13^

Hypertension was the most prevalent risk factor in ischemic stroke affecting 76% of our patients. The findings are in concordance with the results of a study conducted in the South Western community of Nepal and Australia.^14,15^ Among acute ischemic stroke patients with diabetes mellitus, more than half 76% had hypertension and it is higher in comparison to those without diabetes 58.25%. The finding is supported by the previous study which revealed that hypertension proportion has been increasing over time for both with or without diabetes mellitus, but more significantly increased in patients with diabetes mellitus in comparison to those without diabetes mellitus.^16^

Diabetes mellitus in our acute ischemic stroke patients is in poor glycemic control with 44% having HbA1C level >7%. This finding is concomitant with the study in Australia.^15^ However, the study of the USA (64%) and Italy (65.2%) reported a higher prevalence of poorly controlled diabetes mellitus in stroke patients than our study.^15^ This might be due to differences in the size of studies and medication adherence. The poor glycemic control in diabetes mellitus was found to have worsened the functional outcomes; and increased stroke severity, mortality rate, and stroke recurrence.^7^

There are few limitation in our study. Due to the small sample size and selected population from our single centre, the findings of this study can not be generalized to a broader population.

## CONCLUSIONS

The prevalence of diabetes mellitus among acute ischemic stroke patients is higher than other studies done in similar settings. Prompt monitoring of blood glucose levels and early treatment of diabetes mellitus could be done to help lower the morbidity and mortality related to ischemic stroke. Surveillance records should be set up in every health care so that the risk factors of ischemic stroke and their prevalence are recorded.
